# Burden and trends of drug use disorders in young adults: global insights from GBD 2021

**DOI:** 10.3389/fpsyt.2025.1503564

**Published:** 2025-09-24

**Authors:** Xin Su, Teng Fan, Qi Quan, Jun Kan, Zeyu Liu, Bei Zhang, Yuanyuan Huang

**Affiliations:** Sun Yat-sen University Cancer Center, State Key Laboratory of Oncology in South China, Guangdong Provincial Clinical Research Center for Cancer, Guangzhou, China

**Keywords:** age-period-cohort study, drug use disorder, Global Burden of Diseases, opioid use disorders, cannabis use disorders

## Abstract

**Background:**

Drug use disorders (DUDs) is a serious global health crisis, particularly affecting adolescents and young people. The increasing prevalence of DUDs has led to the development of chronic diseases, including cancer, although it has significant impacts on health and life, it is often overlooked in research.

**Method:**

This study utilized GBD 2021 data to assess the burden of four drug use disorders in the young adult population. The data, covering 1991 to 2021, included metrics such as age-sex-year incidence, prevalence, deaths, and disability-adjusted life years (DALYs). Age-standardized rates were used for comparing burden across years and regions, and joinpoint analysis evaluated trends. The Bayesian Age-Period-Cohort model was employed to project future burden. The study also examined the relationship between DUDs burden and socio-economic conditions using the Social Development Index (SDI) and stratified data by WHO regions. Additionally, population attributable fractions were calculated within the GBD comparative risk assessment framework.

**Results:**

Cannabis use disorder (CUDs) emerged as the most prevalent, the ASPR was 617.22/100,000 in 2021. The highest age-standardized mortality rates (ASMR) and age-standardized DALYs rates (ASDR) was observed in OUDs, at (1.46 [1.37-1.55]) and (236.61[185.21-292.47]), respectively. The region of the Americas accounted for the largest proportion of this burden. Opioid use disorders (OUDs) exhibited a notable rising trend, with the age-standardized prevalence rate (ASPR) of 359.62/100,000 in 2021, with a concentration primarily in the European region and the region of the Americas. Male had a higher burden of DUDs than female in the young adults. The burden of DUDs was mainly concentrated under the age of 25, especially CUDs and OUDs. The ASMR and ASDR of DUDs also showed significant growth trends in high SDI areas. Drug use’s contribution to cancer risk, particularly liver cancer due to hepatitis C virus (HCV), had been progressively increasing.

**Conclusions:**

The burden of OUDs and CUDs, continued to escalate annually, especially among young adult males who face heightened risks. Notably, drug use is increasingly contributing to liver cancer mortality and DALYs, emphasizing the urgency of interventions. This study provides evidence for evaluating the burden transfer between different demographic data.

## 1 Introduction

Drug use disorders (DUDs) emphasize the excessive or inappropriate use of substances by individuals, leading to significant negative impacts on physical, psychological, or social functioning (1). This encompasses not only physiological drug dependence but also intense psychological cravings and changes in behavior patterns, affecting an individual’s social functioning, career, and physical health. DUDs primarily encompass opioid use disorders (OUDs), cocaine use disorders (CODs), amphetamine use disorders (AUDs), and cannabis use disorders (CUDs) (1). The harm of uncontrolled drug addiction cannot be ignored. Individuals with DUDs face severe health consequences, including cardiovascular and cerebrovascular diseases, infectious diseases, and mental health disorders (2–5). Additionally, DUDs can lead to family breakdowns, job loss, and increased crime rates, contributing to various social issues (6). According to the 2024 World Drug Report launched by the United Nations Office on Drugs and Crime, the global figure of individuals engaging in drug use escalated to 292 million in 2022, marking a 20% surge from ten years ago. Cannabis remained the most widely used drug worldwide (228 million users), followed by opioids, amphetamines. Many countries and regions’ healthcare and social welfare systems are under tremendous pressure as a result. Among these, OUDs have become a global crisis, particularly in high-income countries. The so-called “Opioid Epidemic”, which began in the late 1990s in North America due to the over-prescription of opioid painkillers, has led to widespread misuse of both prescription and illicit opioids, including fentanyl (7). In the United States alone, opioid overdoses claimed over 80,000 lives in 2023 and This epidemic has since spread to other regions (8). Importantly, adolescents and young adults have not been spared, with increasing rates of opioid misuse observed in this age group (9).

Adolescents and young adults (broadly defined as age 15–39) represent critical windows of vulnerability for the initiation and escalation of substance use (10). Within this spectrum, the subgroup aged 15–25 undergoes rapid neurodevelopmental, emotional, and social transitions, which can heighten susceptibility to risk-taking behaviors (11–13). In many countries, the average age of first drug use is under 18, and early exposure is closely linked to higher risks of developing substance dependence and long-term health consequences. Recent policy shifts, including the legalization of recreational cannabis in parts of North America and Europe, have coincided with a notable increase in cannabis use among youth, who often perceive it as harmless or less risky than other substances (14). For instance, national survey data from the United States show that cannabis use among adolescents significantly increased in states following legalization (15). Similar policies in Europe and Canada mirror this upward trend (14). The increasing prevalence of drug use among young adults contributes to a higher incidence of drug use disorders (DUDs), potentially increasing the risk for various chronic diseases. In particular, adolescence is a critical period for brain maturation and synaptic remodeling. Emerging evidence suggests that repeated exposure to drug use (e.g. cannabinoids) during this period may disrupt endocannabinoid system (ECS) signaling and lead to long-term alterations in dopaminergic and glutamatergic neurotransmission. These neurobiological disruptions may contribute to impaired cognitive control and increased vulnerability to psychiatric comorbidities (16). Beyond mental health impacts, emerging research suggests that DUDs may also contribute to the early onset of various chronic diseases. However, the complex and potentially causal links between DUDs and chronic disease outcomes—particularly cancer—remain understudied. These trends raise urgent concerns about the normalization of substance use among youth, the risk of polysubstance exposure, and the long-term burden of both mental and physical illness, including malignancies.

Given the rising global burden of DUDs, the growing number of affected youths, and the long-term consequences including cancer risk, a comprehensive, age-stratified, and substance-specific evaluation of the global burden of drug use disorders among adolescents and young adults is urgently needed. This study aims to fill that gap by using Global Burden of Disease (GBD) 1991–2021 data to examine time trends, geographic patterns, and future projections of DUDs in youth populations worldwide.

## 2 Methods

### 2.1 Overview and data acquisition

The Global Burden of Diseases, Injuries, and Risk Factors Study (GBD) 2021 is an authoritative study that comprehensively examines global health loss, providing detailed estimates of the widespread impact of 371 diseases and injuries across a global scale, geographical regions, and specifically in 204 countries and regions (17). The framework offers a comprehensive assessment of mortality causes and risk factors worldwide, incorporating intricate classifications by age, gender, geographical location, and socio-demographic index (SDI). Data spanning from 1991 to 2021 on each DUD, namely OUDs, CODs, AUDs, and CUDs, were retrieved from the Global Health Data Exchange’s GBD results tool. This encompassed metrics such as age-sex-year incidence, prevalence, deaths and disability-adjusted life years (DALYs) in terms of counts and crude rate (per 100,000 population) and their 95% uncertainty intervals. Meanwhile, detailed information (deaths and DALY) on the impact of drug use as a risk factor on cancers was also obtained. This study intends to examine the DUDs burden in young adult population (18, 19). The GBD estimates were chosen 15–39 years to obtain data on the DUDs in the younger adult population. For a focused overview of DUDs based on age, we divide people into five groups based on a 5-year age range. Given the potential overlap of conditions in the same individual, the study not provide combined estimates of different DUDs.

### 2.2 SDI estimation and World Health Organization regions

The SDI is a composite indicator of social background and economic conditions that influence health outcomes in each location. SDI is a composite indicator based on estimates of total fertility rate in those younger than 25 years, mean years of education in individuals older than 15 years, and lag-distributed income per capita (20). SDI is used to explore the association between DUDs burden social development degrees in different regions. This index is expressed on a scale of 0-1. An SDI of 0 indicates a theoretical minimum level of development relevant to health, while an SDI of 1 is the theoretical maximum and was used to classify the countries into high, high-middle, middle, low-middle, and low SDI countries (21).

Data was stratified based on the WHO regions, namely Africa, Eastern Mediterranean, Europe, Region of Americas, South-East Asia, and Western Pacific.

### 2.3 Statistical analysis

Age-Standardized Rate (ASR) is aimed at removing the influence of population age structure on overall rates, in order to achieve disease burden comparisons between different years and regions (22). ASR is estimated using the GBD World Population Age Standard as a reference (23). This study used the direct standardization method to calculate the ASR between the ages of 15-39.

The evolution of the disease burden attributed to DUDs was scrutinized employing the joinpoint analysis, which assessed the average annual percent change (AAPC) (24). An upward trend in DUDs burden is signaled by an AAPC value and its corresponding 95% confidence intervals (CI) exceeding zero. Conversely, a declining trend is indicated when both the AAPC value and its 95% CI fall below zero. The Bayesian Age-Period-Cohort framework offers a versatile approach to forecasting disease burden trends. It integrates Bayesian formulas to calculate hypothetical probability distributions constructed from three pivotal dimensions: age, period, and cohort, and cohort and combining *a priori* and sample information to derive posterior information (25). Unlike methodologies solely reliant on sample statistics for parameter estimation, BAPC boasts enhanced flexibility in parameter selection and prior probability distribution specification, thereby producing predictions that are trustworthy. The BAPC model has been shown to have a relatively low absolute percentage bias, so we chose it to predict DUDs burden by 2050 (26). Detailed risk factor estimation methods have been released before GBD 2019 (27). Within the comparative risk assessment framework of GBD, the estimated deaths and DALYs for exposure to risk factors are input to calculate the age-sex-specific population attributable fractions (PAF). Statistical significance was considered at a two-tailed *P* < 0.05. Software packages used in the cause-of-death and risk-factor analysis for GBD 2021 were Stata (version 17), and R (version 4.2.1). Statistical code used for GBD estimation is publicly available online. GBD 2021 study consists of aggregated, de-identified data, and has been approved to waive informed consent with respect to research purposes. Each step used to analyze the GBD 2021 database in the current study followed the guideline in the Guidelines for Accurate and Transparent Health Estimates Reporting (28).

## 3 Results

### 3.1 Global overview

**Figure 1:**
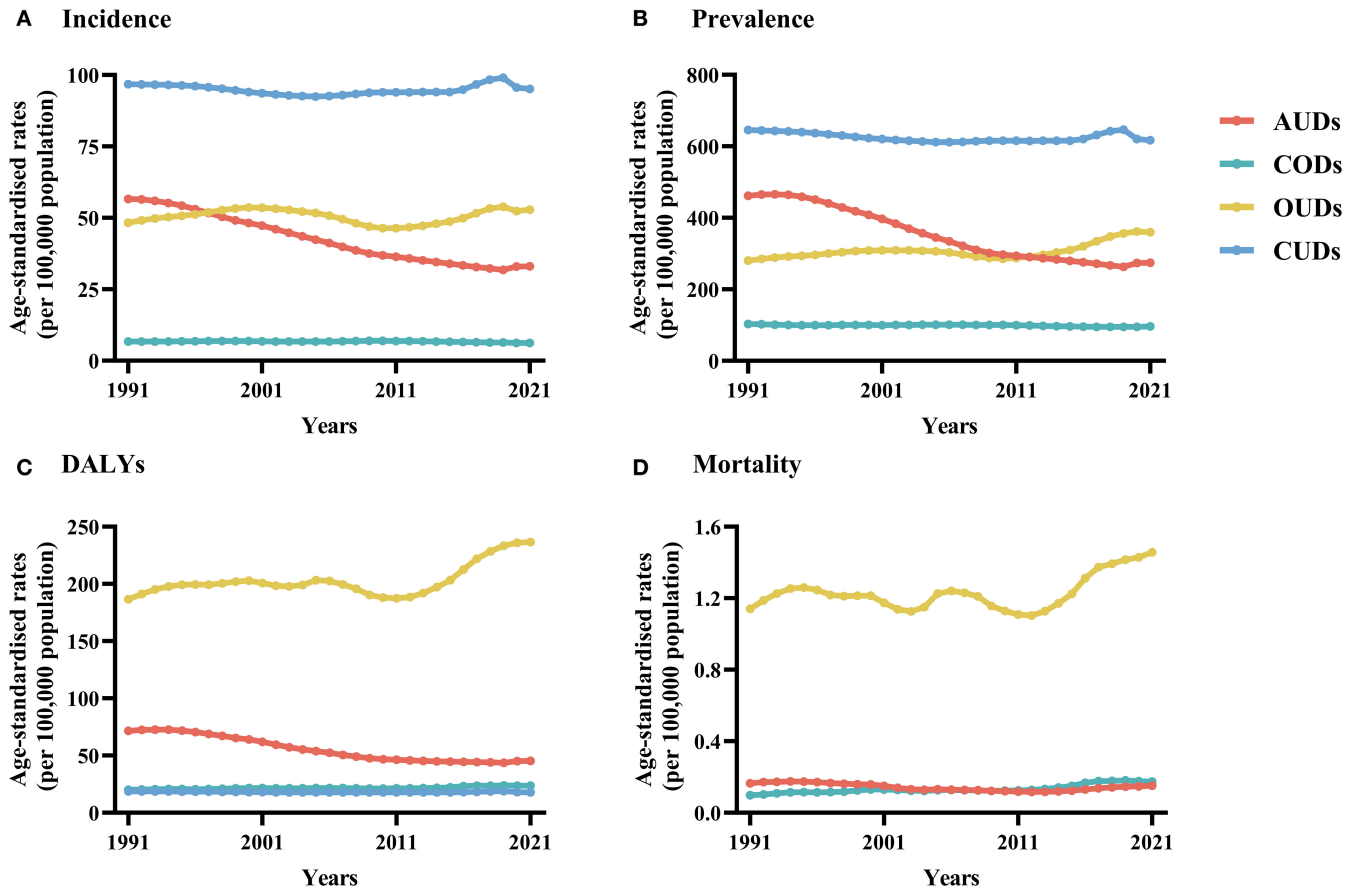
**(A)** Number of incidence and age-standardized incidence rate and **(B)** Number of prevalence and age-standardized prevalence rate and **(C)** Number of DALYs and age-standardized DALYs rate and **(D)** Number of deaths and age-standardized mortality rate in young adult population, at the global level by the four DUDs, 1991–2021. DUDs, drug use disorders; DALYs, disability-adjusted life years. Bar charts depict the total incidence/prevalence/deaths/DALYs and line graphs depict the age-standardized rates of incidence/prevalence/deaths/DALYs. DALYs, disability-adjusted life years.

From 1991 to 2021, the trends of four DUDs among global young adults evolved significantly (**Figure 1**, **Table 1**). OUDs emerged a significant rising trend (AAPC: 0.87, 95% CI: 0.76 to 0.98), with the age-standardized prevalence rate (ASPR) of 359.62/100,000 in 2021. The number of OUD cases increased by nearly 4.6 million over the period. In contrast, CUDs and CODs had declined in prevalence. CUDs emerged as the most prevalent, but the ASPR decreased from 645.76 to 617.22/100,000, while CODs fell from 103.21 to 96.23/100,000. Although the decrease in AUDs was the largest (AAPC: -1.81, 95% CI: -1.94 to-1.67), with a decrease of about 2.8 million cases, it remained higher than that of CODs. Regarding age-standardized incidence rate (ASIR), OUDs showed an increasing trend, rising from 48.33 in 1991 to 52.89 in 2021. The ASIR of AUDs was 33.05 in 2021, with an AAPC of -1.99 (95% CI: -2.11 to -1.88). The ASIR of CUDs slightly decreased, showing a relatively stable trend. In comparison, CODs had the lowest age-standardized incidence rate at 6.23/100,000, with a stable annual decline. The age-standardized mortality rates (ASMR) for CODs and OUDs had significantly increased, with AAPCs of 2.05 (95% CI: 1.54 to 2.57) and 0.96 (95% CI: 0.5 to 1.43), respectively. The ASMR due to AUDs slightly decreased from 0.16 to 0.15/100,000. DALYs are powerful tools for evaluating the overall burden of diseases, taking into account not only the shortened lifespan caused by diseases, but also the long-term impact of diseases on patients’ quality of life. Between 1991 and 2021, the decline in AUDs was significant, with age-standardized DALYs rate (ASDR) decreasing from 71.57 to 45.27/100,000. Notably, the burden of OUDs significantly intensified, as reflected in the substantial rise of DALYs from 186.59 to 236.61/100,000, accompanied by an AAPC of 0.87 (95% CI: 0.45 to 1.29). The ASDR of CUDs and CODs showed slightly changes decreased.

**Table 1:** The global burden of four DUDs from 1991 to 2021.

Element	All-age counts		Age-standardized rate (per 100,000 population)		AAPC	
	1991	2021	1991	2021	*P*	Value
AUDs						
Prevalence	10,523,607.94 (7,296,857.53-14,689,874.11)	8,115,747.98 (5,454,399.37-11,490,928.73)	461.82 (320.24-645.22)	274.01 (184.06-387.84)	<0.001	-1.81 (-1.94 to-1.67)
Incidence	1,324,361.4 (844,701.96-1,936,614.35)	964,542.18 (585,264.25-1,454,527.14)	56.64 (35.98-83.11)	33.05 (20.11-49.73)	<0.001	-1.99 (-2.11 to-1.88)
Deaths	3,675.86 (3,050.71-4,568.55)	4,568.37 (4,090.67-5,163.57)	0.16 (0.14-0.20)	0.15 (0.14-0.17)	0.412	-0.29 (-0.97-0.4)
DALYs	1,629,074 (1,004,507.63-2492032.86)	1,345,631.44 (855,635.31-2,035,888.61)	71.57 (44.17-109.51)	45.27 (28.73-68.55)	<0.001	-1.61 (-1.72 to -1.50)
CUDs						
Prevalence	14,706,851.56 (9,563,848.89-22,156,042.78)	18,168,318.39 (11,833,287.01-27,668,094.7)	645.76 (419.96-972.06)	617.22 (401.84-940.29)	<0.001	-0.3 (-0.41 to -0.19)
Incidence	2,232,978.20 (1,232,006-3,555,496.58)	2,772,822.15 (1,502,722.35-4,510,538.23)	96.77 (53.17-154.63)	95.11 (51.74-154.31)	<0.001	-0.26 (-0.29 to -0.23)
Deaths	–	–	–	–	–	–
DALYs	425,602.85 (231,119.23-729,396.35	525,604.95 (282,647.52-896,372.75)	18.68 (10.14-31.97)	17.86 (9.6-30.48)	<0.001	-0.3 (-0.42 to -0.19)
CODs						
Prevalence	2,310,815.52 (1,581,344.09-3,250,788.74)	2,849,239.87 (2,062,902.26-3,867,811.45)	103.21 (70.79-144.84)	96.23 (69.60-130.79)	<0.001	-0.28 (-0.36 to -0.19)
Incidence	156,301.72 (90,816.9-257,338.49)	179,223.04 (109,672.04-289,663.27)	6.67 (3.86-11.02)	6.23 (3.83-10.05)	<0.001	-0.56 (-0.70 to -0.41)
Deaths	2,110.87 (1,762.66-2,645.26)	5,299.57 (4,880.76-5,886.21)	0.1 (0.08-0.12)	0.17 (0.16-0.19)	<0.001	2.05 (1.54-2.57)
DALYs	447165.52 (308,887.42-646,412.9)	705,904.14 (540,067.10-933,619.84)	20.15 (13.99-29)	23.61 (18.01-31.33)	<0.001	0.57 (0.43-0.71)
OUDs						
Prevalence	6,179,597.51 (4,727,446.44-8,025,405.24)	10,777,814.88 (8,764,688.97-13,360,936.81)	280.56 (215.13-363.94))	359.62 (292.21-446.06)	<0.001	0.87 (0.76-0.98)
Incidence	1,104,487.49 (762,551.83-1,538,981.35)	1,550,954.97 (1,120,791.42-2,073,096.2)	48.33 (33.19-67.66)	52.89 (38.31-70.51)	0.186	0.13 (-0.06-0.33)
Deaths	24,580.34 (21,635.57-27016.16)	44,138.12 (41,422.97-47,074.53)	1.14 (1.01-1.25)	1.46 (1.37-1.55)	<0.001	0.96 (0.50-1.43)
DALYs	4,089,346.63 (3,115,466.68-5182692.55)	7,111,529.87 (5,572,628.74-8,783,839.37)	186.59 (142.50-236.09	236.61 (185.21-292.47)	<0.001	0.87 (0.45-1.29)

OUDs, opioid use disorders; CODs, cocaine use disorders; AUDs, amphetamine use disorders; CUDs, cannabis use disorders; AAPC, average annual percent change; DALYs, disability-adjusted life years.

### 3.2 Gender and age-specific differences in the burden of DUDs

**Figure 2:**
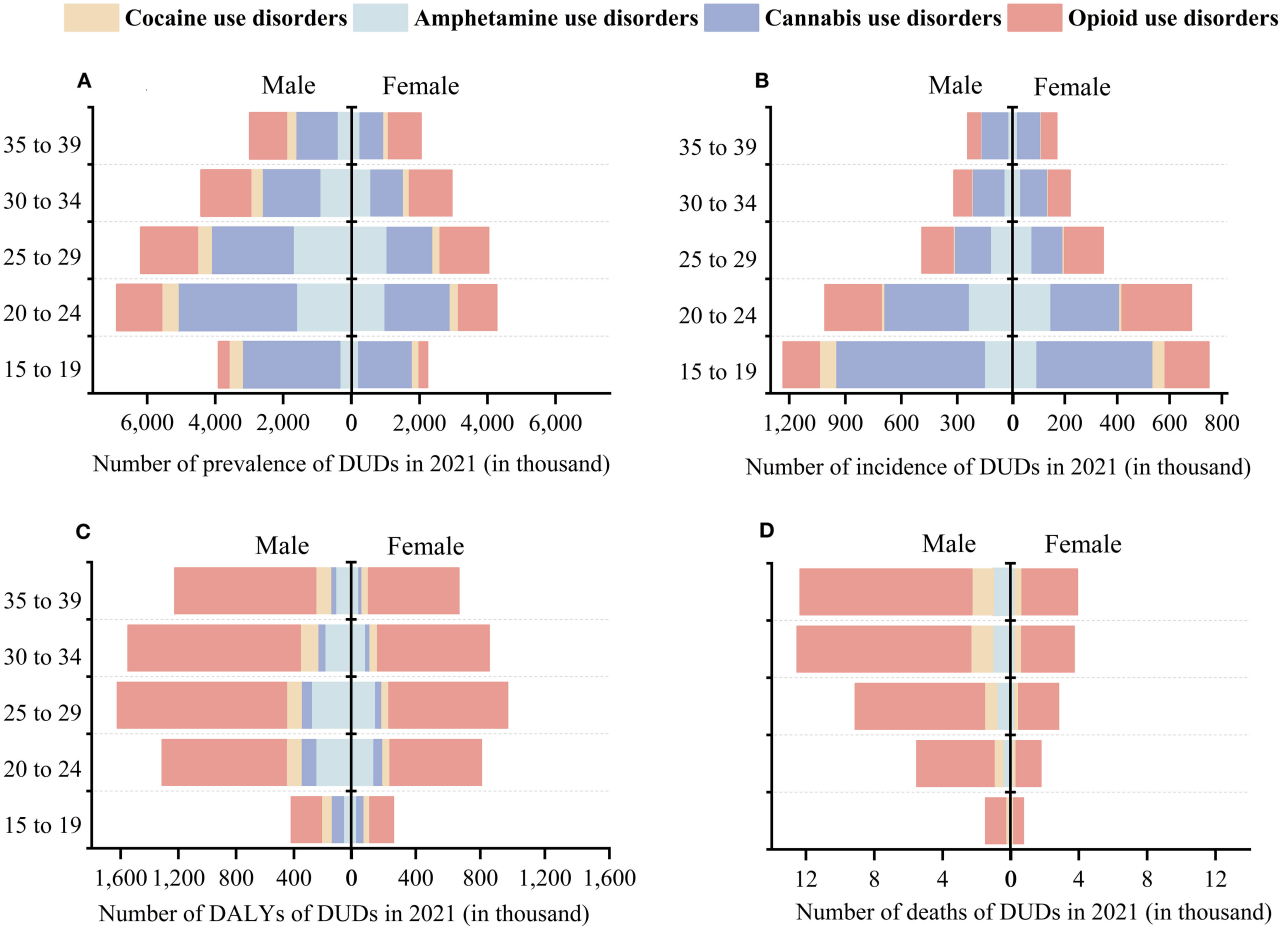
**(A)** Number of prevalence and **(B)** Number of incidence and **(C)** Number of DALYs and **(D)** Number of deaths in the four DUDs, at the global level by sex and age, 1991-2021. DALYs, disability-adjusted life years.

In terms of prevalence, AUDs generally declined across all age groups and genders, with women showing a greater decrease than men (**Figure 2**). In contrast, the prevalence of CUDs remained relatively stable across age groups, albeit with some fluctuations (**Supplementary Table 1**). CODs also declined slowly across age groups and genders, with consistently higher rates in males, though the decline trend of CODs in men aged 30–39 years was slightly greater than that in women. However, OUDs had significantly increased over the past thirty years, with men experiencing faster growth rates overall compared to women. The data showed that the age group of 25 to 29 had the highest crude rate, with males at 554.73 and females at 492.08/100,000 (**Supplementary Table 1**). And the AAPC in the age group of 35–39 was the highest, with females at 0.93 (95% CI: 0.79 to 1.07) and males at 1.02 (95% CI: 0.94 to 1.11).

Regarding the incidence of DUDs, we observed that the overall trend for AUDs showed a gradual decline, with the magnitude of the decline diminishing as the age increases (**Figure 2**). Among them, the group aged 15 to 24 had the largest decrease in AUDs (AAPC: -2.02, 95% CI: -2.15 to -1.89), with females showing a greater decrease than males (**Supplementary Table 2**). The incidence rate of CUDs in males was higher than that in females across all age groups in 2021 (**Supplementary Table 2**). And the incidence of CUDs was the highest among the four DUDs, especially in the age groups of 15–19 and 20-24 (248.48 and 149.63/100,000). CODs had the lowest crude rate among the four DUDs. Notably, the 15–19 age group showed a higher incidence rate of 21.56/100,000 compared to other age groups, with males experiencing higher rates than females (15.35 and 27.45/100,000, respectively). Among young adults, the incidence of OUDs in females was gradually rising. For individuals under 30, both genders exhibited increasing incidence rates, with the highest crude rate found in the 20–24 age group: 99.6 for males and 90.42/100,000 for females (**Supplementary Table 2**).

**Figure 3:**
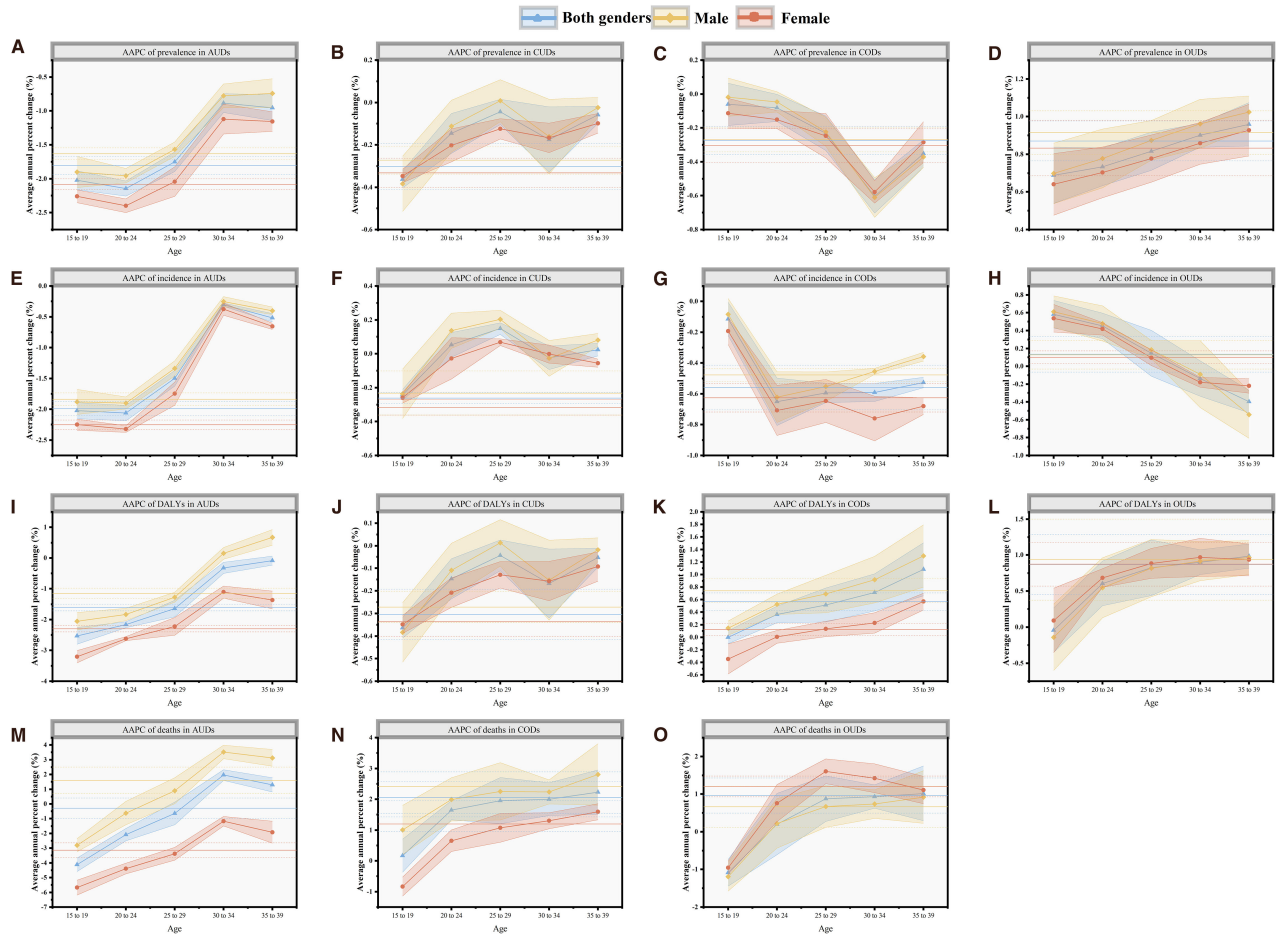
**(A–D)** the AAPC of prevalence in four DUDs; **(E–H)** the AAPC of incidence in four DUDs; **(I–L)** the AAPC of DALYs in four DUDs; **(M-O)** the AAPC of DALYs in three DUDs, except CUDs at global by sex and age, 1991-2021. The solid horizontal line represents the AAPC in the 15–39 age group, and the dashed horizontal line represents the 95% confidence intervals of AAPC. OUDs, opioid use disorders; CODs, cocaine use disorders; AUDs, amphetamine use disorders; CUDs, cannabis use disorders; AAPC, average annual percent change.

From 1991 to 2021, the number of deaths related to AUDs among women decreased, showing a downward trend across all age groups (**Figure 2**). During the same period, the crude rate of men due to AUDs increased from 0.15/100,000 to 0.23/100,000, with an AAPC of 1.6 (0.69 - 2.51), indicating a significant upward trend (**Figure 3**). Mortality rates for men fell in the 15–24 age group but rose for those over 25. The mortality rates related to CODs in both genders showed an upward trend across all age groups (**Figure 3**). Additionally, the number of deaths related to OUDs surpassed other DUDs. Men experienced a higher burden of OUD-related mortality compared to women (33,467.11 vs. 10,671), with experiencing upward trends in mortality rates (**Figure 3**). Among these, women in the 20–24 age group showed a downward trend, while the downward trend among men was observed in the 30–34 age group.

In terms of DALYs, the burden for AUDs decreased among young adults, with males showing higher DALYs than females (58.43 vs. 31.64). Males had increasing DALYs for AUDs after age 30, whereas females saw a decrease in the 15–39 age range (**Figure 3**). From 1991 to 2021, the burden of DALYs for CUDs slightly decreased, with a higher crude rate in the 20–24 age group, and was greater in males across all age groups.

The DALYs of male and female CODs showed an increasing trend, and the magnitude increased with age. The burden on men was also higher than that on women (**Supplementary Table 4**). The DALYs burden for OUDs was the highest, peaking in the 25–29 age group for both genders, with men experiencing a greater burden than women (**Figure 3**).

### 3.3 DUDs burden differences based on country and GBD region

**Figure 4:**
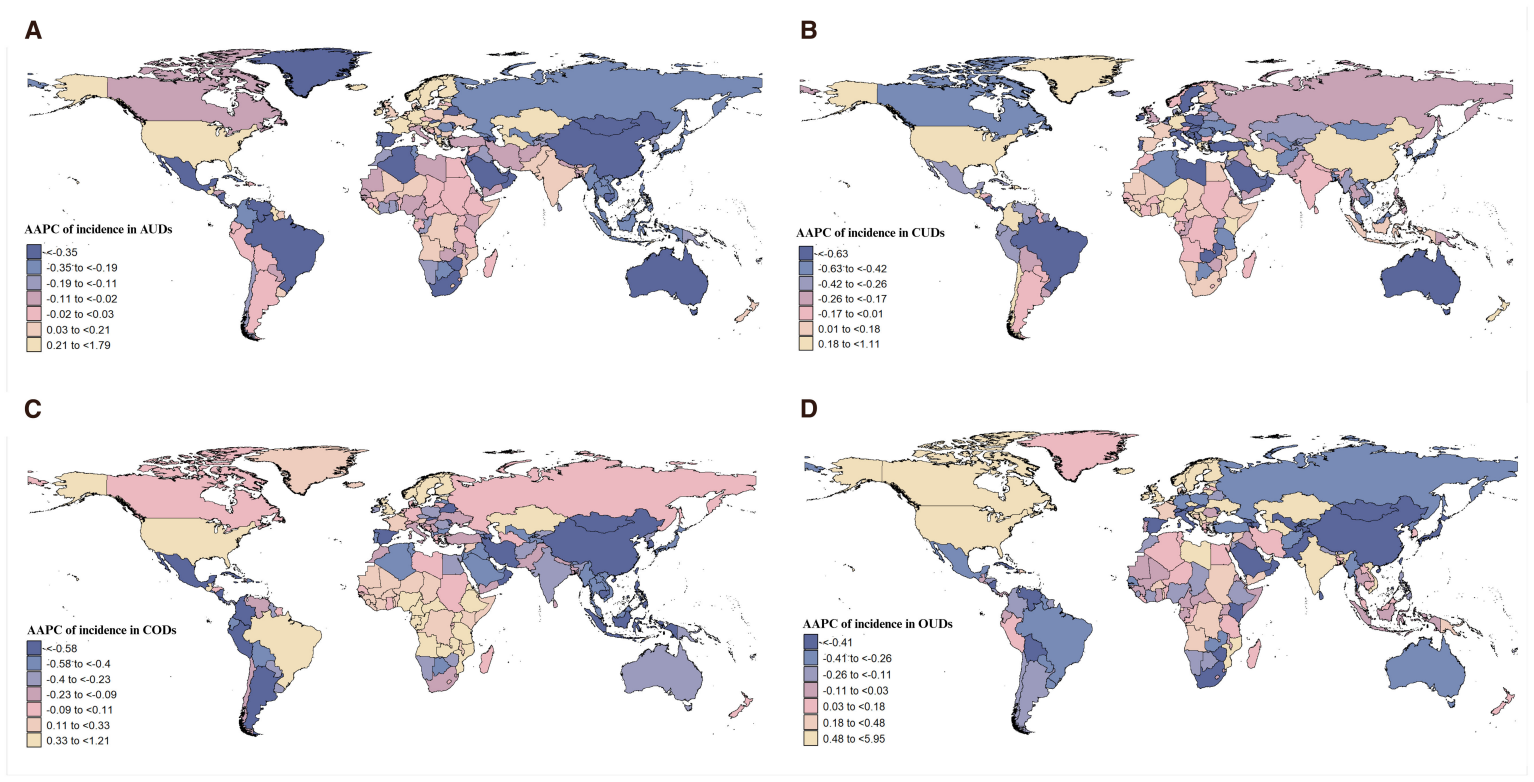
The AAPCs of incidence in **(A)** AUDs, **(B)** CUDs, **(C)** CODs and **(D)** OUDs globally.

As shown in **Figure 4** and **Supplementary Figure 1**, from 1991 to 2021, Sweden exhibited the highest AAPC in the incidence and prevalence of AUDs at 1.79 (95% CI: 1.55 to 2.03), while Mauritius had the highest AAPC in deaths at 20.96 (95% CI: 18.48 to 23.48). In terms of DALYs, the United States of America had the highest annual average growth rate. Notably, China demonstrated the largest annual average decline across all four measures (**Supplementary Figure 3**). Australia experienced the most significant annual average decline in the incidence, prevalence, and DALYs of CUDs, whereas Colombia saw the highest annual average growth globally (**Supplementary Figures 1**, **3**). Regarding CODs, Mauritius had the highest annual average growth rate in deaths and DALYs globally (**Supplementary Figures 2**, **3**). Mozambique and Estonia were shown the highest average annual growth rates in incidence and prevalence, respectively. Conversely, China recorded the most significant decline across all four measures. The United States of America led globally in the annual average growth rate in incidence, prevalence, and DALYs of OUDs in the past 30 years, ranking third only in terms of deaths attributed to OUDs (**Figure 4**, **Supplementary Figures 1**–**3**).

**Figure 5:**
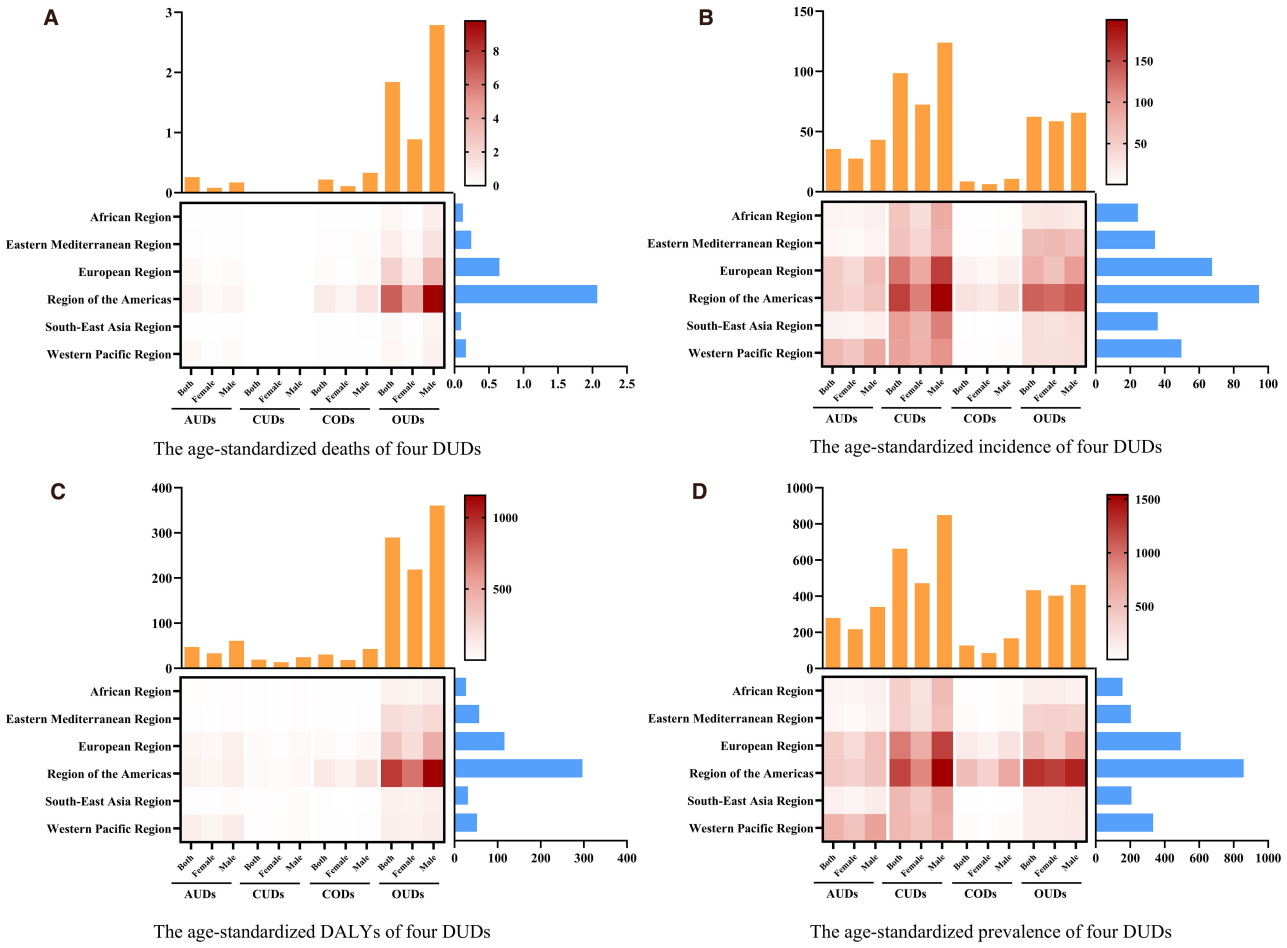
The trends of age-standardized rate of **(A)** mortality, **(B)** incidence **(C)** DALYs and **(D)** prevalence in DUDs by GBD regions in 2021.

A horizontal comparison of the disease burden of the four DUDs among young adults in 2021 revealed that CUDs had the highest ASIR and ASPR, with a concentration primarily in the European region and the region of the Americas, shown as **Figure 5**. Notably, the burden on males was higher than on females, and OUDs followed CUDs in terms of disease burden. In terms of DALYs and mortality, OUDs carried a heavier burden than the other DUDs. The region of the Americas accounted for the largest proportion of this burden (**Figure 5**).

### 3.4 Burden of DUDs with socio-demographic index

**Figure 6:**
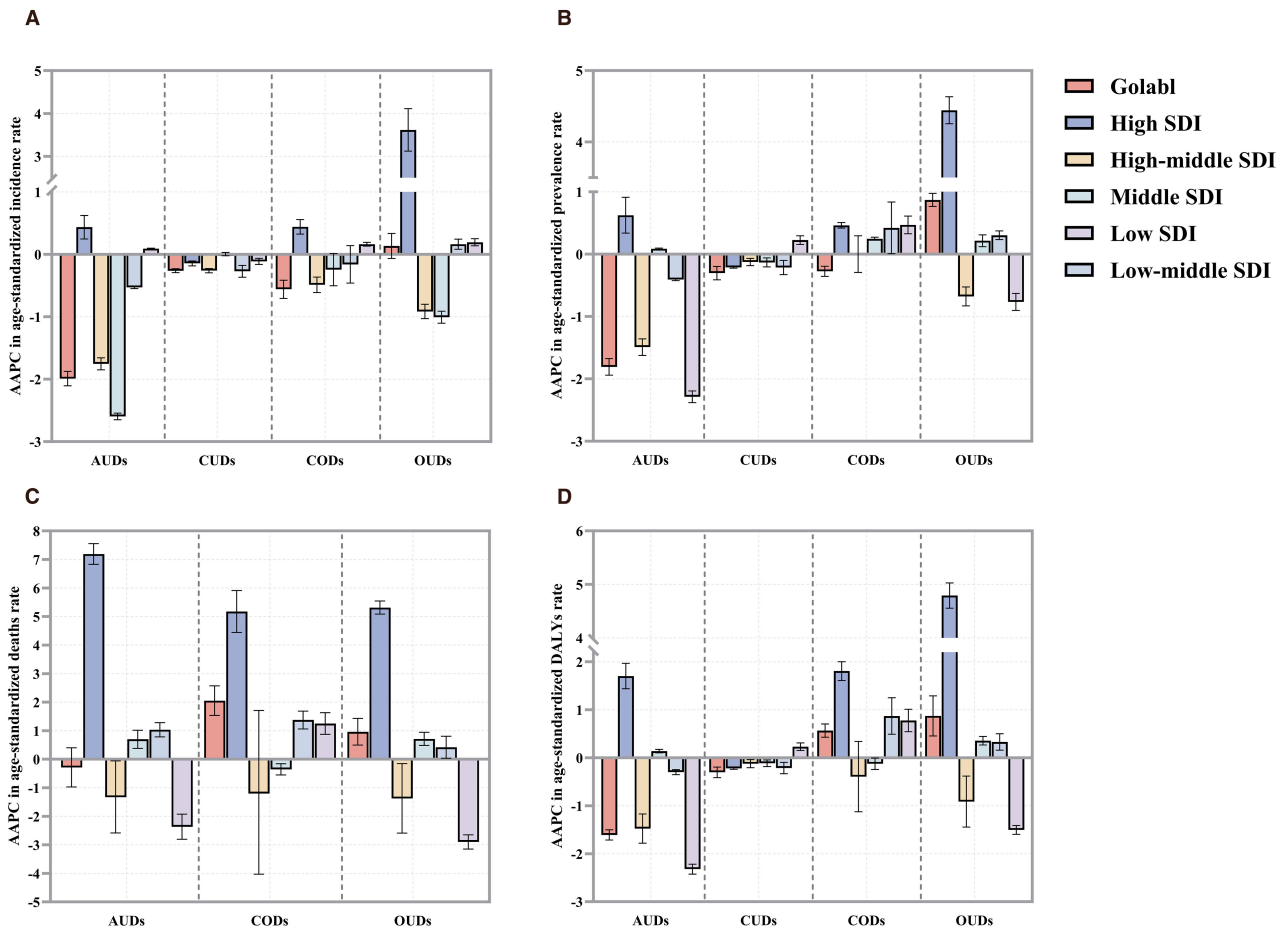
The AAPCs in age-standardized rates of **(A)** incidence, **(B)** prevalence **(C)** deaths, and DALYs **(D)** due to DUDs from 1991 to 2021 by global and SDI quintile. SDI, socio-demographic Index.

Globally, from 1991 to 2021, the prevalence, incidence, and DALYs related to AUDs demonstrated a declining trend. Nevertheless, in high-SDI countries, there was a contrary trend of increasing incidence, prevalence, mortality, and DALYs associated with AUDs (**Figure 6**). From 1990 to 2019, CUDs showed a downward trend in all four aspects at both the global and SDI levels. The prevalence of CODs only showed an increasing trend in high SDI countries, but in terms of mortality and DALYs, it was gradually increasing in low-middle SDI countries and low SDI countries (**Figure 6**). Between 1991 and 2021, OUDs exhibited a rapid progression in terms of prevalence, incidence, mortality, and DALYs, compared to other DUDs. Conversely, four measures of low SDI countries remained stability or even showed a declining trend during this period (**Figure 6**).

### 3.5 Predictive analysis on DUDs burden to 2050

**Figure 7:**
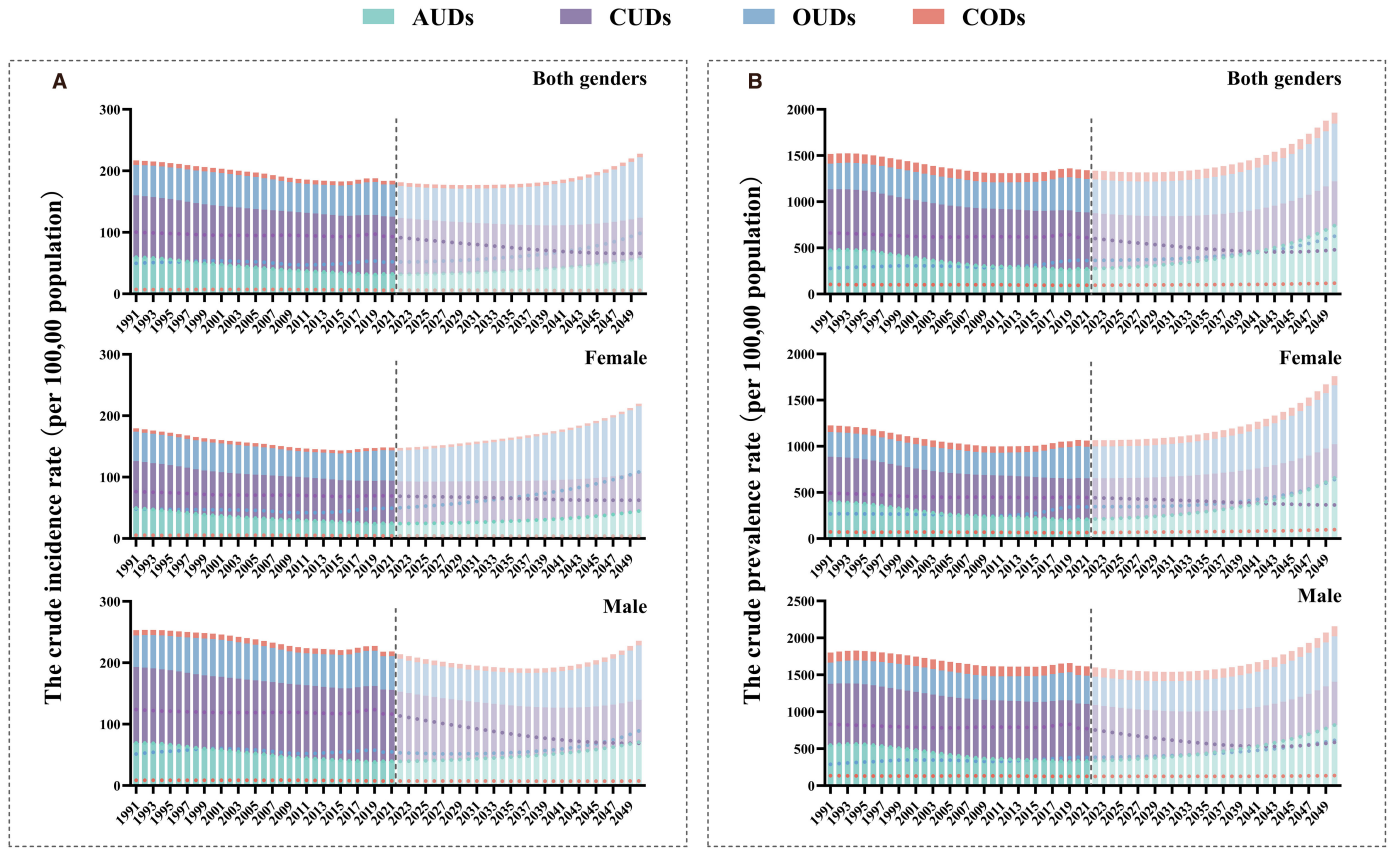
The predicted crude rate of four DUDs burden by sex to 2050 in global. **(A)** The predicted crude rate of incidence to 2050; **(B)** The predicted crude rate of prevalence to 2050.

The predicted crude rate of incidence, prevalence of four DUDs to 2050 were illustrated in **Figure 7**. Globally, the crude rates of incidence and prevalence of AUDs and OUDs were predicted to increase to 2050. The prognosis indicated a reduction in both the crude rates of incidence and prevalence of CUDs by the year 2050.The predicted incidence rate of CODs exhibited a declining trend among female, whereas it showed an upward trend in male.

### 3.6 The impact of drug use as a risk factor for cancers

**Figure 8:**
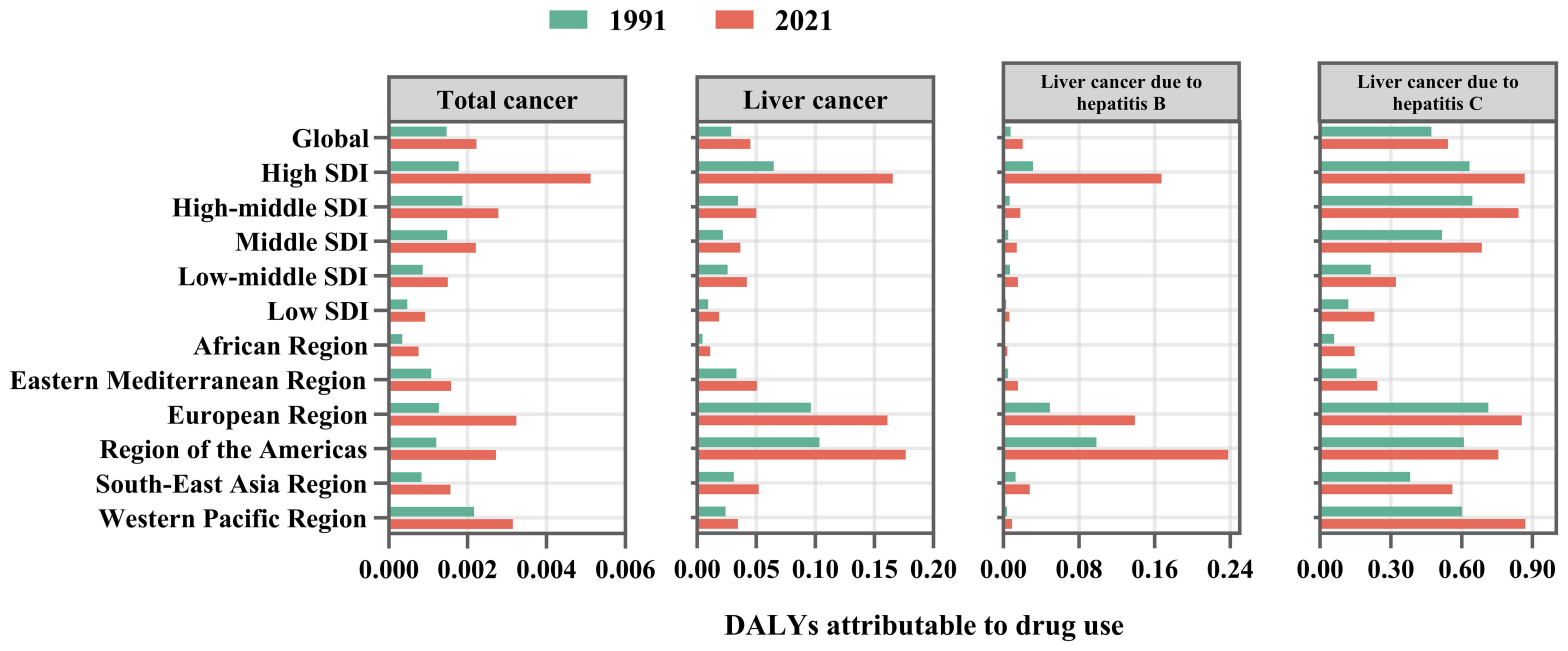
The PAFs of mortality of drug use on cancers in 2021 by GBD region and SDI level. PAF, population attributable fraction.

We employed the GBD tool to conduct a thorough analysis of the specific impact of drug use on the burden of cancer determined that it primarily focuses on the burden of liver cancer, particularly liver cancer due to hepatitis B and liver cancer due to HCV. In 1991, drug use was associated with a higher disease burden of total cancers (**Figure 8**). According to the population attributable fraction, drug use has a greater impact on DALYs and mortality rates among men with liver cancer, especially in the high SDI countries, European region and region of the Americas(**Supplementary Tables 13**, **14**). In 2021, the impact of drug use on global DALYs and mortality had increased, particularly in the case of l liver cancer due to HCV (**Figure 8**). Furthermore, we have observed a substantial rise in the PAF of mortality and DALYs over the past 30 years in countries with high SDI. The contribution of drug use to Liver cancer due to HCV was substantial, particularly in the Western Pacific Region in 2021 (**Figure 8**).

## 4 Discussion

A comprehensive analysis of DUDs from 1991 to 2021 revealed a complex and dynamic evolution of these conditions among young adults, particularly across spatial, temporal, and gender dimensions. This section elucidates the major observed trends, focusing on shifting burdens across demographics and their implications for public health strategies.

The period under review saw a notable increase in OUDs among young adults globally. In 2021, there were approximately 10.78 million prevalent cases and 1.55 million incident cases of OUDs. The data indicated a significant rise in prevalence and incidence, particularly pronounced in the 25–29 age group, which was consistent with previous regional studies. A previous study highlighted early initiation of marijuana use as a significant predictor of subsequent OUD risk (29). This alarming trend highlighted the growing public health crisis associated with opioid misuse. The increase in OUDs in this age group may be attributed to a range of factors including prescription practices, socio-economic stressors, and the availability of opioids (30, 31). Given the substantial rise in OUD cases, it is imperative to enhance preventive measures and treatment options specifically targeting this vulnerable age group. Conversely, AUDs generally declined among younger populations, with the most pronounced decrease observed in the 15–24 age group. This trend could reflect successful public health interventions, drug substitution, or shifts in social norms. CUDs exhibited relative stability in prevalence, albeit with age-specific variations. The highest incidence rates were observed in the 15–24 age group, indicating an early age of onset for CUDs. In 2021, over 9.8 million individuals aged 15–24 were affected by CUDs, pointing to persistent challenges in curbing cannabis use. Effective strategies should focus on both prevention and treatment, with particular emphasis on early intervention for adolescents. Recent reports have also emphasized the increasingly younger age of onset for CUDs (32, 33). Recent research has increasingly highlighted the role of the ECS—a key neuromodulatory network involved in reward processing, stress response, and emotional regulation—in the development and maintenance of CUD (34). Dysregulation of ECS signaling, particularly the overactivation of CB1 receptors, has been implicated in heightened susceptibility to cannabis dependence and withdrawal symptoms (35). These neurobiological findings add an important dimension to our understanding of CUDs. Furthermore, targeting ECS-related pathways may offer promising therapeutic strategies (36). The ASIR and age-standardized prevalence of CODs showed a gradual decline, maintaining consistently lower levels compared to other DUDs. The declining trend in CODs may reflect the effectiveness of intervention strategies or changing patterns of drug use. However, the lower prevalence of CODs does not diminish the need for continued vigilance and targeted interventions, especially in regions where cocaine use remains a concern, such as the United States of America.

Gender differences in DUDs were pronounced, underscoring the need for tailored public health approaches. The data from 1991 to 2021 revealed that CODs and CUDs had increased more rapidly among men than women, with men experiencing higher crude rates and a more pronounced growth. In contrast, the decline in AUDs had been more significant among women compared to men. Women showed greater reductions in both the prevalence and incidence of AUDs. Despite these improvements, the burden of AUDs remained higher among men, particularly in terms of mortality and DALYs. This persistent burden among men underscores the need for ongoing efforts to address substance use and its associated health impacts. The rising incidence of OUDs among younger females, particularly in the 20–24 age group, was noteworthy. While the overall burden of OUDs was still higher among men, the increasing rates among women suggested emerging trends that warranted further investigation. Understanding the underlying factors that contributed to this rise in female OUD cases was essential for developing targeted interventions. This may be related to the fact that women are more likely to experience mental disorders, are more susceptible to violence, and are more likely to be prescribed psychotropic medications during pregnancy (37–39). This gender disparity may have been influenced by a range of factors, including differences in drug-seeking behaviors, access to treatment, and societal norms. Addressing the gender-specific needs in DUDs treatment and prevention programs was crucial to effectively mitigate the impact of drug use.

Significant regional variations in the burden of DUDs among young adults were observed, reflecting differences in drug use patterns and the influence of local policies. The Region of the Americas had the highest incidence rates of CUDs, while the United States experienced the most significant growth in OUDs. High-SDI areas experienced an upward trend in the ASIR of AUDs, in stark contrast to the global decline. Additionally, the ASMR and ASDR of DUDs also exhibited significant increases in more developed areas. In contrast, countries with lower SDI scores experienced more stable or declining trends in the ASIR of DUDs, which may reflect different drug use patterns, cultural attitudes or the influence of emerging public health initiatives (40, 41). The varying trends across regions highlighted the need for tailored strategies that address local drug use patterns and socio-economic conditions. In addition, forecasts to 2050 indicate that the number of OUD cases among young adults will continue to increase. The increasing burden of DUDs among young males also warrants further attention.

Our analysis revealed that drug use’s contribution to cancer risk among young adults, particularly liver cancer related to HCV, has been progressively increasing over the past 30 years. This trend was especially pronounced in the Western Pacific Region and among men. The rising PAF for both DALYs and mortality. This growing burden may be partly attributed to injection drug use, which substantially increases the risk of HCV transmission—a key etiological factor in liver cancer. Individuals who inject drugs account for the majority of HCV virus infections, with prevalence estimates ranging from 50% to 90% (42). Persistent HCV virus infection can lead to chronic hepatic inflammation, progressive fibrosis, and ultimately cirrhosis, creating a pro-tumorigenic microenvironment. In addition to injection-related transmission, non-injection drug use has also been associated with a higher prevalence of HCV infection. A systematic review of 28 studies reported HCV prevalence among individuals who engage in non-injection drug use ranging from 2.3% to 35.3% (43). The findings indicated that HCV prevalence in individuals who engage in non-injection drug use is substantially higher than in the general population. However, the underlying mechanisms of transmission in this group remain unclear.

Combined models of care, incorporating addiction treatment, HCV management, and psychosocial support, are more effective than siloed approaches. Policymakers and healthcare providers should prioritize the expansion of such integrated care frameworks, particularly in regions with high DUD and HCV burdens, to mitigate the progression to liver cancer and improve long-term health outcomes.

Despite previous reports on the limitations of the GBD (44, 45), it remains necessary to clarify the limitations specific to this study. Although the GBD Study 2021 has improved models to enhance estimation accuracy, certain cross-cutting limitations remain. Sparse data from specific regions and time periods may affect estimate accuracy; for example, data quality and coverage remain poor in South Asia. Additionally, due to the lack of estimates for deaths caused by CUDs in the GBD 2021 data, the total number of deaths attributed to DUDs may be underestimated.

## 5 Conclusions

The burden of DUDs has been escalating annually, showing significant differences between countries and SDI levels, posing a severe challenge to global health systems and social stability. Heightening awareness of the hazards associated with drug abuse and evidence-based treatments is crucial, with a particular focus on OUDs and CUDs, which disproportionately affect young adult males. Notably, drug use is increasingly contributing to cancer mortality and DALYs, particularly for liver cancer, underscoring the urgency of intervention. Effective public health strategies must address the disparities in prevalence, gender differences, and regional variations, with a focus on tailored prevention and treatment efforts to mitigate the impact of DUDs and improve public health outcomes in the future. We call on policymakers and all healthcare professionals to collaborate in addressing this global health challenge, optimizing resource allocation, and strengthening prevention and treatment measures to control and alleviate the heavy burden of DUDs.

## Data Availability

The original contributions presented in the study are included in the article/**Supplementary Material**. Further inquiries can be directed to the corresponding author.
