# Supplementary material for: Burden and trends of drug use disorders in young adults: global insights from GBD 2021
Source: Front Psychiatry. 2025 Sep 24;16:1503564. doi: 10.3389/fpsyt.2025.1503564 (PMC12504863; doi:10.3389/fpsyt.2025.1503564)
Supplement: Supplementary file 1 [file Supplementaryfile1.zip › Supplementary Figures.DOCX]

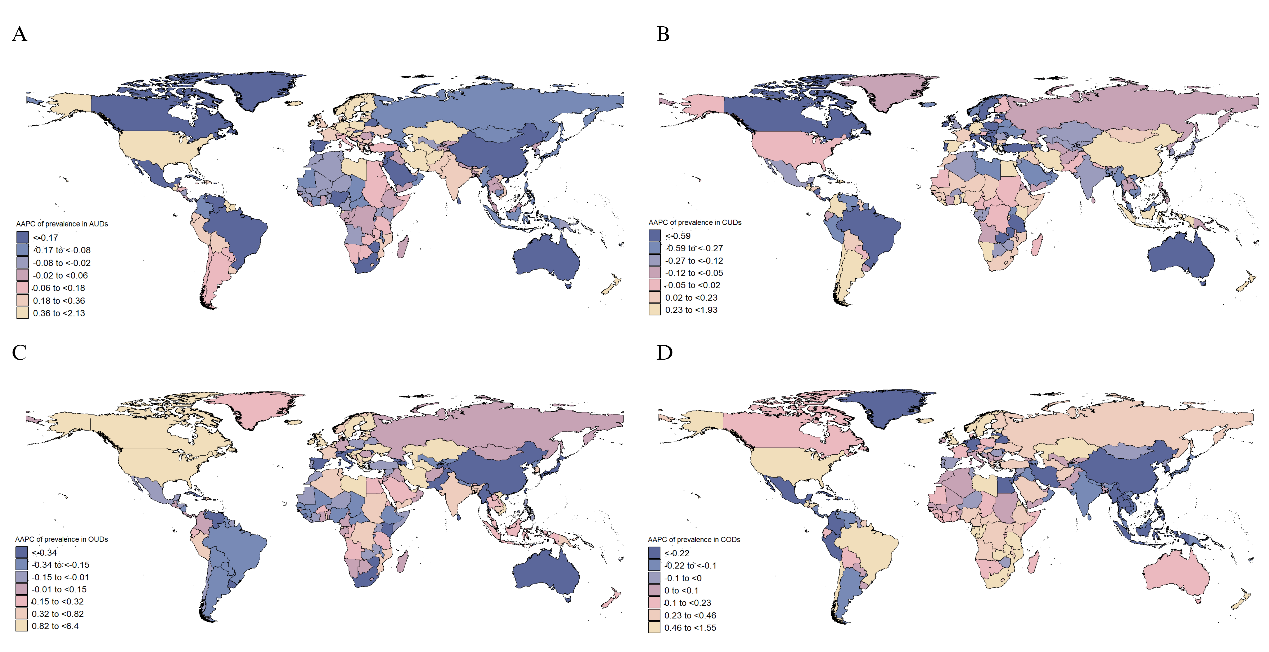
 Supplementary Figure 1. The AAPCs of prevalence in A) AUDs, B) CUDs, C) CODs and D) OUDs globally. OUDs, opioid use disorders; CODs, cocaine use disorders; AUDs, amphetamine use disorders; CUDs, cannabis use disorders; AAPC, average annual percent change; DALYs, disability-adjusted life years.


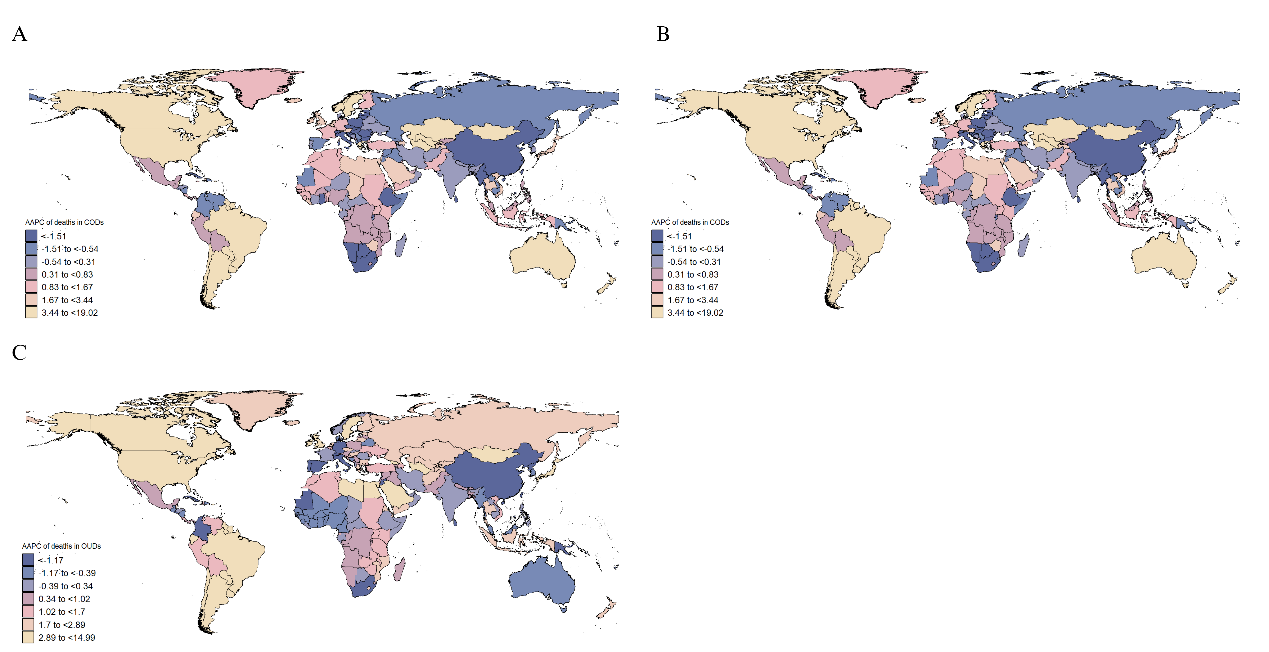


Supplementary Figure 2. The AAPCs of morality in A) AUDs, B) CUDs, C) CODs and D) OUDs globally.
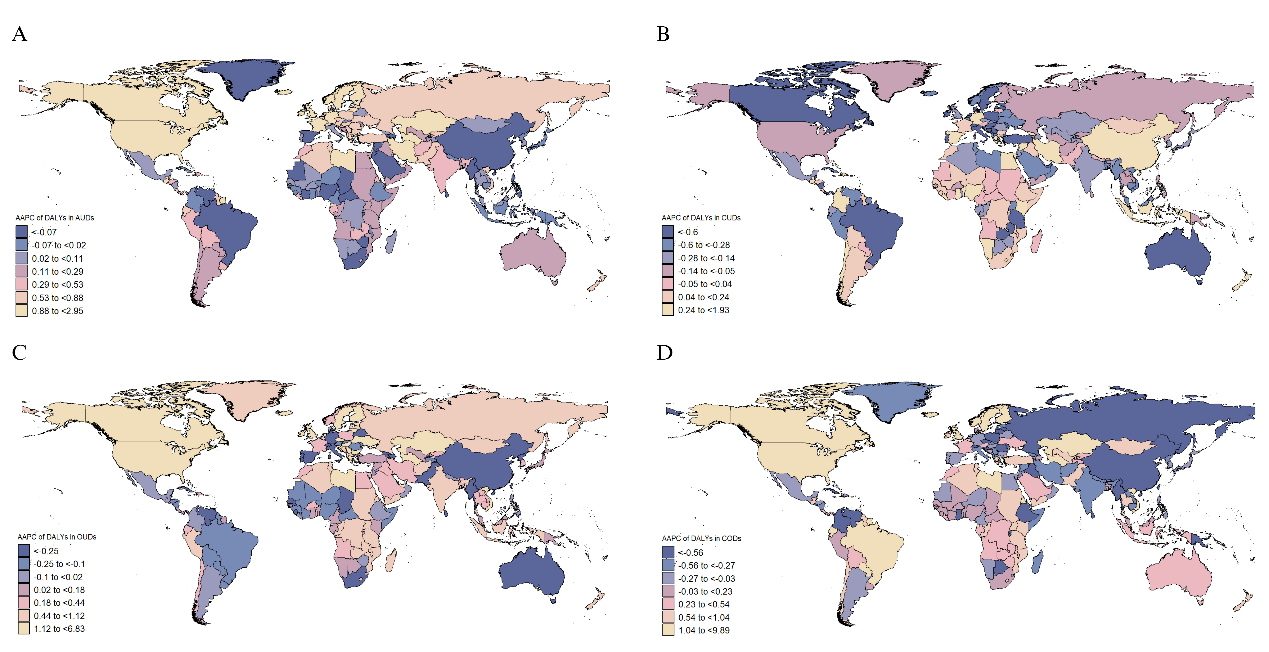


Supplementary Figure 3. The AAPCs of DALYs in A) AUDs, B) CUDs, C) CODs and D) OUDs globally.
